# Global Estimates of Ambient Fine Particulate Matter Concentrations from Satellite-Based Aerosol Optical Depth: Development and Application

**DOI:** 10.1289/ehp.0901623

**Published:** 2010-03-16

**Authors:** Aaron van Donkelaar, Randall V. Martin, Michael Brauer, Ralph Kahn, Robert Levy, Carolyn Verduzco, Paul J. Villeneuve

**Affiliations:** 1 Department of Physics and Atmospheric Science, Dalhousie University, Halifax, Nova Scotia, Canada; 2 Harvard-Smithsonian Center for Astrophysics, Cambridge, Massachusetts, USA; 3 School of Environmental Health, University of British Columbia, British Columbia, Canada; 4 NASA Goddard Space Flight Center, Greenbelt, Maryland, USA; 5 Dalla Lana School of Public Health, University of Toronto, Toronto, Ontario, Canada; 6 Population Studies Division, Health Canada, Ottawa, Ontario, Canada

**Keywords:** aerosol, aerosol optical depth, AOD, particulate matter, PM_2.5_

## Abstract

**Background:**

Epidemiologic and health impact studies of fine particulate matter with diameter < 2.5 μm (PM_2.5_) are limited by the lack of monitoring data, especially in developing countries. Satellite observations offer valuable global information about PM_2.5_ concentrations.

**Objective:**

In this study, we developed a technique for estimating surface PM_2.5_ concentrations from satellite observations.

**Methods:**

We mapped global ground-level PM_2.5_ concentrations using total column aerosol optical depth (AOD) from the MODIS (Moderate Resolution Imaging Spectroradiometer) and MISR (Multiangle Imaging Spectroradiometer) satellite instruments and coincident aerosol vertical profiles from the GEOS-Chem global chemical transport model.

**Results:**

We determined that global estimates of long-term average (1 January 2001 to 31 December 2006) PM_2.5_ concentrations at approximately 10 km × 10 km resolution indicate a global population-weighted geometric mean PM_2.5_ concentration of 20 μg/m^3^. The World Health Organization Air Quality PM_2.5_ Interim Target-1 (35 μg/m^3^ annual average) is exceeded over central and eastern Asia for 38% and for 50% of the population, respectively. Annual mean PM_2.5_ concentrations exceed 80 μg/m^3^ over eastern China. Our evaluation of the satellite-derived estimate with ground-based *in situ* measurements indicates significant spatial agreement with North American measurements (*r* = 0.77; slope = 1.07; *n =* 1057) and with noncoincident measurements elsewhere (*r* = 0.83; slope = 0.86; *n =* 244). The 1 SD of uncertainty in the satellite-derived PM_2.5_ is 25%, which is inferred from the AOD retrieval and from aerosol vertical profile errors and sampling. The global population-weighted mean uncertainty is 6.7 μg/m^3^.

**Conclusions:**

Satellite-derived total-column AOD, when combined with a chemical transport model, provides estimates of global long-term average PM_2.5_ concentrations.

Chronic exposure to airborne fine particulate matter with diameter < 2.5 μm (PM_2.5_) is associated with adverse human health impacts including morbidity and mortality (e.g., Dockery et al. 1993; McDonnell et al. 2000; Pope et al. 2009). Several national environmental agencies in North America and Europe monitor PM_2.5_ concentrations at numerous sites throughout their jurisdictions, but even these relatively dense networks have limited geographic coverage. Few long-term measurement sites exist elsewhere in the world, particularly in rapidly developing countries where concentrations and estimated health impacts are greatest (Cohen et al. 2004). Point measurements collected at monitoring sites are not necessarily representative of regional concentration, and regional variability is difficult to assess from point measurements alone. In recent years, application of satellite observation to surface air quality has advanced considerably (Hoff and Christopher 2009; Martin 2008). In fact, global aerosol observations from satellite remote sensing could substantially improve estimates of population exposure to PM_2.5_.

Since the mid 2000s, the MODIS (Moderate Resolution Imaging Spectroradiometer) and MISR (Multiangle Imaging Spectroradiometer) instruments onboard the National Aeronautics and Space Administration’s (NASA) Terra satellite has provided global observations of aerosol optical depth (AOD), a measure of light extinction by aerosol in the atmospheric column above the earth’s surface. Terra’s sun-synchronous orbit encircles the earth approximately 15 times each day, with each pass crossing the equator at approximately 1030 hours local solar time. Observations of AOD from Terra provide daily insight into the global distribution of column-integrated aerosol. However, the applicability of AOD to surface air quality depends on several factors, including the vertical structure, composition, size distribution, and water content of atmospheric aerosol.

Many studies have investigated the relationship between total-column AOD and surface PM_2.5_ measurements. Most have developed simple empirical relationships between these two variables (e.g., Engel-Cox et al. 2004a; Wang and Christopher 2003); more recent investigations often have used local meteorological information to better relate AOD and PM_2.5_ (e.g., Koelemeijer et al. 2006; Liu et al. 2005) or to filter the AOD (e.g., Gupta et al. 2006). Some studies have employed light detection and ranging (LIDAR) instruments to capture the vertical aerosol distribution at specific locations (e.g., Engel-Cox et al. 2006; Schaap et al. 2008). Schaap et al. (2008) noted that locally derived AOD–PM_2.5_ relationships cannot be extended easily to other regions because of variation in meteorology and aerosol composition. Unique, local, time-dependent AOD–PM_2.5_ relationships are necessary to infer global estimates of PM_2.5_. Ground-based measurements of aerosol vertical profiles and properties have insufficient coverage to estimate global AOD–PM_2.5_ relationships.

Global chemical transport models (CTMs) resolve atmospheric composition at a resolution of hundreds of kilometers horizontally by hundreds of meters vertically, with a temporal frequency of tens of minutes. Liu et al. (2004) first estimated surface-level PM_2.5_ from MISR observations by using CTM output to represent local AOD–PM_2.5_ conversion factors over the contiguous United States. van Donkelaar et al. (2006) extended the approach used by Liu et al. (2004) to estimate PM_2.5_ from both MODIS and MISR observations and investigated the factors affecting the agreement between AOD and surface-level PM_2.5_. Statistical models have also been used to relate AOD to PM_2.5_. For example, Liu et al. (2007) used MISR-retrieved spherical versus nonspherical particle fraction, in addition to model-derived vertical distribution, to separate mineral dust from other aerosol species. More recently, Paciorek and Liu (2009) probed the limitations of using AOD without accounting for vertical distribution or speciation and concluded that agreement with ground-based monitors based on this approach might depend on factors other than satellite observations.

We developed a global satellite-based estimate of surface PM_2.5_ at a spatial resolution of 0.1° × 0.1°, or approximately 10 km × 10 km at midlatitudes. We developed an approach for combining MODIS and MISR AOD into a single improved estimate of AOD. Using this methodology, we calculated AOD–PM_2.5_ conversion factors with a global CTM and produced and applied these factors to the AOD. We present a global estimate of PM_2.5_ concentrations and validate it with ground-based (*in situ*) observations. We estimate global exposure to outdoor ambient PM_2.5_ using our satellite-derived product to demonstrate potential application for global health studies. We then examined sources of error.

## Materials and Methods

### Satellite observations

The MODIS instrument measures a wide range of spatial and spectral information from its orbit aboard the Terra satellite. The near-daily global coverage from the MODIS AOD retrieval (Levy et al. 2007) is advantageous due to frequent measurements. The MISR instrument (Diner et al., 1998), which is also on board Terra, offers smaller spatial and spectral ranges, but views each scene on the earth from nine different angles. This additional angular information allows the MISR AOD retrieval (Diner et al. 2005; Martonchik et al. 2009) to reduce algorithmic assumptions and retrieval bias, as well as obtain information about microphysical properties and plume heights in aerosol source regions (Kahn et al. 2007). Neither instrument can retrieve AOD in cloudy conditions.

We used the MODIS BRDF/Albedo product (MOD43, Collection 5; Schaaf et al. 2002) to distinguish surface types, in conjunction with ground-based retrievals of AOD, and to identify regions of high bias in both MODIS and MISR AOD. We defined these surface types for each month according to the ratio of surface albedo for different wavelengths, similar to assumptions inherent in the MODIS AOD retrieval. We removed AOD that was retrieved from either instrument with an anticipated bias greater than the larger of ± (0.1 or 20%), based on comparison with the Aerosol Robotic Network (AERONET; Holben et al. 1998) sun photometer measurements of AOD. Remaining MODIS and MISR AODs were averaged to produce a single value at a given grid cell. The Supplemental Material (doi:10.1289/ehp.0901623) describes in detail the satellite retrievals and this bias filtration. We restricted our subsequent analysis to locations with at least 50 successful satellite retrievals for 2001–2006 to yield a nearly complete (95%) global geographic coverage.

### Estimating PM_2.5_ from AOD

Estimating ground-level concentrations of dry 24-hr PM_2.5_ (micrograms per cubic meter) from satellite observations of total-column AOD (unitless) requires a conversion factor that accounts for their spatially and temporally varying relationship:


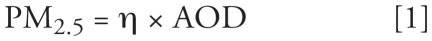


η is a function of the factors that relates 24-hr dry aerosol mass to satellite observations of ambient AOD: aerosol size, aerosol type, diurnal variation, relative humidity, and the vertical structure of aerosol extinction (van Donkelaar et al. 2006). Following the methods of Liu et al. (2004, 2007) and van Donkelaar et al. (2006), we used a global 3-D CTM [GEOS-Chem; geos-chem.org; see Supplemental Material (doi:10.1289/ehp.0901623)] to calculate the daily global distribution of η.

The GEOS-Chem model solves for the temporal and spatial evolution of aerosol (sulfate, nitrate, ammonium, carbonaceous, mineral dust, and sea salt) and gaseous compounds using meteorological data sets, emission inventories, and equations that represent the physics and chemistry of atmospheric constituents. The model calculates the global 3-D distribution of aerosol mass and AOD with a transport time step of 15 min. We applied the modeled relationship between aerosol mass and relative humidity for each aerosol type to calculate PM_2.5_ for relative humidity values that correspond to surface measurement standards [European Committee for Standardization (CEN) 1998; U.S. Environmental Protection Agency 1997] (35% for theUnited States and Canada; 50% for Europe). We calculated daily values of η as the ratio of 24-hr ground-level PM_2.5_ for a relative humidity of 35% (U.S. and Canadian surface measurement gravimetric analysis standard)and of 50% (European surface measurement standard) to total-column AOD at ambient relative humidity. We averaged the AOD between 1000 hours and 1200 hours local solar time, which corresponded to the Terra overpass period. We interpolated values of η from 2° × 2.5°, the resolution of the GEOS-Chem simulation, to 0.1° × 0.1° for application to satellite AOD values.

We compared the original MODIS and MISR total-column AOD with coincident ground-based measurements of daily mean PM_2.5_. Canadian sites are part of the National Air Pollution Surveillance Network (NAPS) and are maintained by Environment Canada (http://www.etc.cte.ec.gc.ca/NAPS/index_e.html). The U.S. data were from the Interagency Monitoring of Protected Visual Environments (IMPROVE) network (http://vista.cira.colostate.edu/improve/Data/data.htm) and from the U.S. Environmental Protection Agency Air Quality System Federal Reference Method sites (http://www.epa.gov/air/data/index.html). Validation of global satellite-derived PM_2.5_ estimates was hindered by the lack of available surface-measurement networks in many parts of the world. To supplement this lack of available surface measurements, we collected 244 annually representative, ground-based PM_2.5_ data from both published and unpublished field measurements outside the United States and Canada[see Supplemental Material (doi:10.1289/ehp.0901623)].

## Results

**Figure 1:**
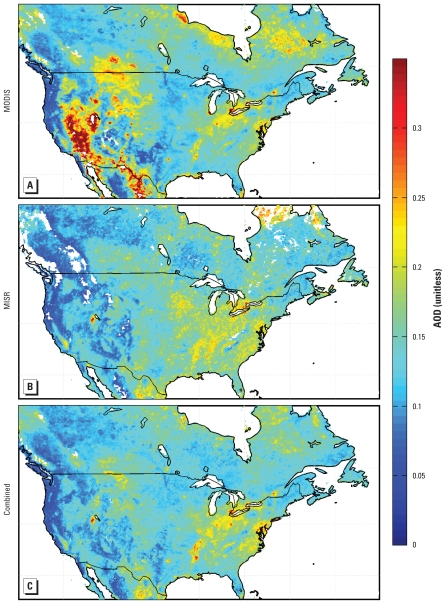
Mean AOD for 2001–2006 from the MODIS (*A*) and MISR (*B*) satellite instruments. (*C*) Data from the combined product developed here. White space denotes water or < 50 measurements.

In Figure 1A and 1B, we show the mean AOD for retrievals from 1 January 2001 to 31 December 2006 over North America from MODIS and MISR. Both data sets exhibit similar AOD values of 0.15–0.25 over the eastern United States, which reflect a combination of anthropogenic and biogenic sources. Several individual cities can be clearly identified in mean MODIS AOD for the Great Lakes region. A large AOD enhancement over the southwestern United States appears in the MODIS retrievals but is absent from the MISR retrievals. Figure 1C presents the mean combined MODIS and MISR AODs over North America. Our filtration of these two AOD products removes the biased AOD observed by MODIS over the western United States. The combined product is dominated by MODIS in the east because of finer temporal sampling. MISR dominates in the west because of its accuracy.

In Table 1, we provide statistics that compare the spatial variation in 6-year mean AOD retrievals with measurements of daily 24-hr average PM_2.5_ sampled on the same days as successful satellite observations. Both the MODIS and MISR instruments indicate some relationship between retrieved total-column AOD and *in situ* PM_2.5_, both with spatial correlation coefficients of 0.39. A simple average of the daily AOD from both instruments yields a correlation of 0.44. Combining retrievals from these instruments as described in the Methods section increases the correlation to 0.61. Additional information is required to quantitatively estimate PM_2.5_ concentrations from AOD, as presented below.

**Figure 2:**
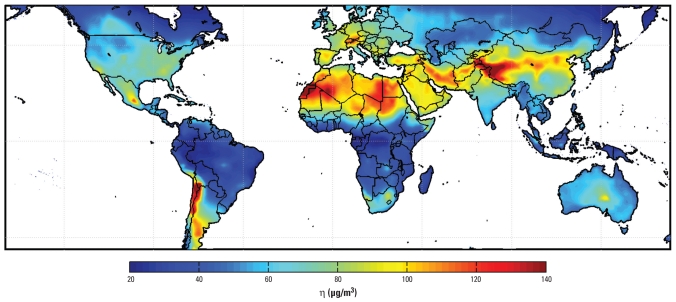
Annual mean η (ratio of PM_2.5_ to AOD) for 35% relative humidity. White space indicates water.

Figure 2 shows the annual mean distribution of daily η values used to relate satellite-observed total-column AOD to PM_2.5_ at 35% relative humidity. Average values of η are typically 20–130 μg/m^3^. High values of η over regions with large dust concentrations (Prospero et al. 2002) reflect, in part, the low hygroscopicity of dust. Values of η are lower for hygroscopic aerosols, as their dry volume is significantly smaller than under ambient conditions. Ground-level aerosol sources in industrial regions lead to vertical profiles that peak near ground and to moderate values of η. Western North America is characterized by low η, which provides additional insight into the poor AOD–PM_2.5_ correlations (Engel-Cox et al. 2004b; Hu 2009) associated with this region and in agreement with Liu et al. (2007), who found that transported dust aloft affects the western North America AOD–PM_2.5_ relationship. η is related to land types only insofar as these are typified by particular aerosol types, meteorology, and vertical structures. Temporal variation in η is considerable.

**Figure 3:**
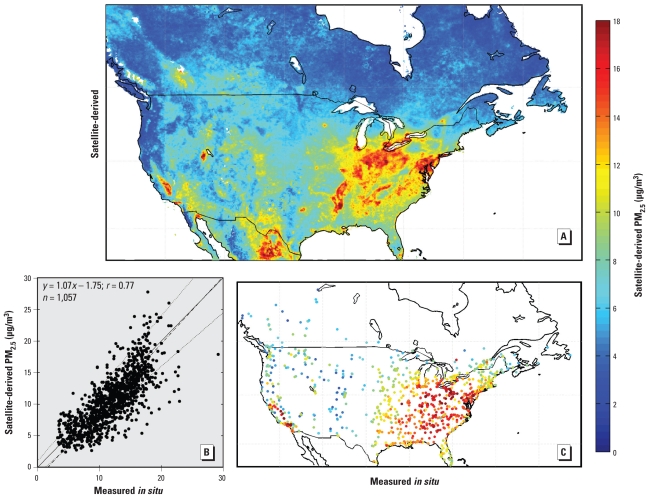
Satellite-derived PM_2.5_ and comparison with surface measurements. (*A*) Mean satellite-derived PM_2.5_ between 2001 and 2006; white space denotes water or < 50 AOD measurements. (*B*) Average coincident values of both measured and satellite-estimated PM_2.5_. The solid black line denotes unity; thin dotted lines show uncertainty of ± (1 μg/m^3^ + 15%); and the dashed line represents the best fit (Hirsh and Gilroy 1984). (*C*) Positions and mean values of coincidently measured surface sites.

Figure 3A shows the 6-year mean of 24-hr average satellite-derived surface PM_2.5_ over North America as calculated from Equation 1 at a daily time scale. A large-scale PM_2.5_ enhancement is apparent over the eastern United States. The western and northern parts of the continent are generally characterized by low concentrations, with a few exceptions. Geographic mean PM_2.5_ concentrations over eastern and western North America are 6.9 μg/m^3^ and 6.2 μg/m^3^, respectively. Application of η (Figure 2) increased the spatial contrast relative to Figure 1, which reflects ground-level aerosol sources in the east and aerosols aloft in the north and west.

We evaluated the satellite-derived PM_2.5_ with surface monitors. Figure 3C shows the annual mean of 24-hr PM_2.5_ concentrations measured with the surface monitors and sampled on the same days as the satellite-derived PM_2.5_. Ground-level measurements show features similar to our satellite-derived product. Figure 3B quantitatively compares satellite-derived and ground-level measured PM_2.5_. We found significant cross-sectional correlation between average coincidently sampled satellite-derived and ground-based PM_2.5_ across North America (*r* = 0.77; slope = 1.07; bias = −1.75 μg/m^3^). Many factors contribute to the scatter of points, including differences between what satellite and *in situ* measurements represent, that do not necessarily indicate errors in either measurement.

### Global estimates of PM_2.5_ concentrations

**Figure 4:**
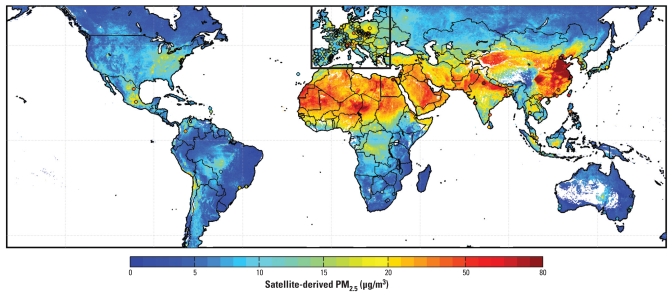
Global satellite-derived PM_2.5_ averaged over 2001–2006. White space indicates water or locations containing < 50 measurements. Circles correspond to values and locations of comparison sites outside Canada and the United States; the black box outlines European sites.

In Figure 4, we present the 6-year mean of our global satellite-derived PM_2.5_. This figure, and all subsequent figures, are at 50% relative humidity, which is in agreement with European ground-based measurements. We rejected points created with < 50 values, enabling 95% global geographic coverage. The satellite-derived PM_2.5_ include an adjustment for discontinuous sampling, as described in the error analysis. The annual mean PM_2.5_ concentrations vary spatially by more than an order of magnitude. Values are < 10 μg/m^3^ for large regions of the earth. In contrast, PM_2.5_ concentrations of 60–90 μg/m^3^ are found over eastern China, with values > 100 μg/m^3^ for its major industrial regions. The Indo-Gangetic plain, from New Delhi eastward contains the highest PM_2.5_ concentrations in India, with values of 80–100 μg/m^3^, especially in winter (e.g., Di Girolamo et al. 2004). Concentrations elsewhere in northern India are 15–60 μg/m^3^. The effects of biomass burning on PM_2.5_ levels are visible in central South America and central Africa, where we estimated concentrations of 10–17 μg/m^3^. Dust transport in the fine mode is substantial (Jones and Christopher 2007) and contributes to large-scale PM_2.5_ of approximately 20–50 μg/m^3^ in the Middle East.

Figure 4 also shows locations of ground-based measurements and values outside North America that were used for comparison. Despite increased uncertainty because of temporal sampling differences, significant overall agreement exists (*r* = 0.83; slope = 0.86; intercept = 1.15 μg/m^3^; *n* = 244). Similar agreement is obtained when all sites except Europe and North America are considered (*r* = 0.83; slope = 0.91; intercept = −2.64 μg/m^3^; *n* = 84).

**Figure 5:**
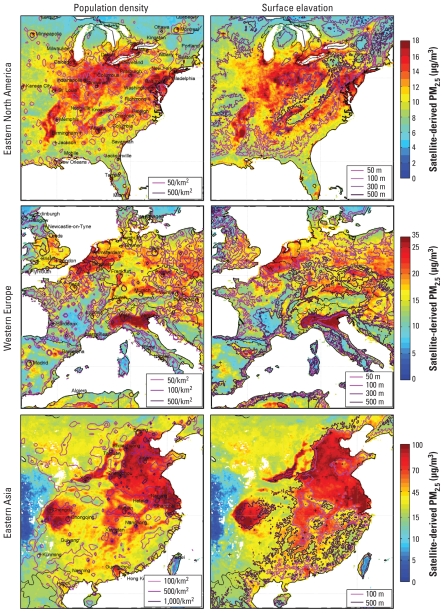
Regional satellite-derived PM_2.5_ concentrations. Columns show mean satellite-derived PM_2.5_ for 2001–2006 at locations that contain at least 50 measurements. Contours denote population density (left) and surface elevation (right). Crosses indicate city centers. Note the different color scales for each region. Altitude data are from the U.S. Geological Survey (1996).

Figure 5 overlays contours of population density and surface elevation onto satellite-derived PM_2.5_ for regions of major anthropogenic sources: eastern North America, western Europe, and eastern Asia. Some relationships are apparent between PM_2.5_, topography and population. Heavily populated and highly polluted, low-lying regions of eastern China and the Po Valley of northern Italy contrast sharply with neighboring higher altitude regions. The Appalachian Mountains in eastern North America emerged as a relatively clean region. Many PM_2.5_ enhancements were associated with urban or industrial areas, but these relationships are complex.

### Error analysis

The dominant sources of error in satellite-derived PM_2.5_ arose from uncertainties in both AOD retrieval and aerosol vertical structure (van Donkelaar et al. 2006). The residual AOD bias after data filtering is within the larger of ± (0.1 or 20%), as evaluated with ground-based AERONET measurements. We evaluated the GEOS-Chem simulation of the aerosol vertical profile using observations from the Cloud-Aerosol LIDAR and Infrared Pathfinder Satellite Observation (CALIPSO) satellite (Winkler et al. 2007). The GEOS-Chem simulation generally captures to within 5% the fraction of AOD within the boundary layer [see Supplemental Material (doi:10.1289/ehp.0901623)]. We estimate the error in satellite-derived PM_2.5_ as the change in PM_2.5_ that occurs when η and AOD are adjusted by their uncertainty, approximated as the GEOS-Chem vertical profile bias and residual satellite AOD bias, respectively.

**Figure 6:**
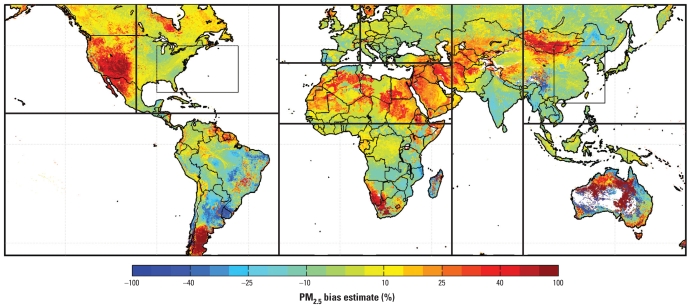
Estimate of the satellite-derived PM_2.5_ bias, defined as (satellite-derived PM_2.5_ – truth) ÷ truth. Boxed areas outline the regions used in Figure 8; see Supplemental Material (doi:10.1289/ehp.0901623) for the two subregions.

Figure 6 shows the error distribution of coincidently sampled satellite-derived PM_2.5_. Arid regions are typically overpredicted and populated regions of East Asia underpredicted. We found that 1 SD of the global error distribution is within ± 15% of the satellite-derived value. We tested this uncertainty estimate by comparing coincident PM_2.5_ observations for North America (Figure 2) and find that 1 SD of the data lies within ± (1 μg/m^3^ + 15%). The necessary inclusion of a small absolute term suggests that our uncertainty estimate may be underestimated at low PM_2.5_ values and supports the presence of a small negative bias (Figure 3B).

**Figure 7:**
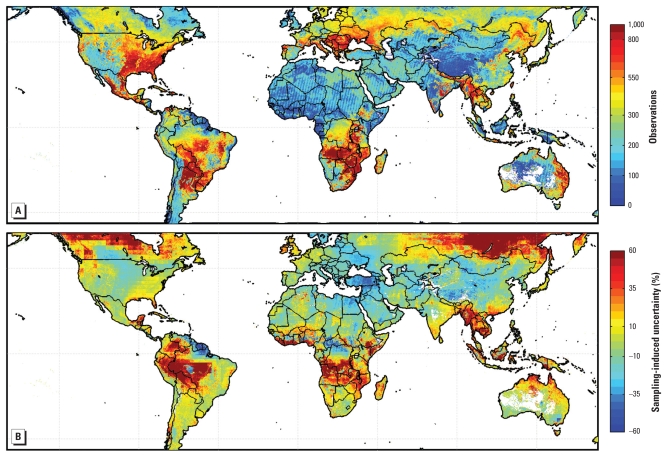
Satellite-derived PM_2.5_ sampling and its estimated induced uncertainty. (*A*) Total number of values used from satellite per 0.1° grid box. (*B*) Percentage change in average coincidently sampled simulated PM_2.5_ concentrations relative to a full-year average.

Nonuniform and incomplete sampling by satellites have the potential to create bias in long-term mean observations (Levy et al. 2009; Paciorek and Liu 2009). Here we investigate how nonrandom sampling of AOD by satellite observations affects the representation of annual mean PM_2.5_. The total number of successful satellite retrievals are shown in Figure 7A and are summarized regionally as population-weighted mean in Table 2. Lower sampling was fortuitously collocated with lower population. The global population-weighted mean of observations per 0.1° × 0.1° box was 297. The percent difference between a GEOS-Chem simulation of PM_2.5_ sampled coincidently with daily satellite-derived PM_2.5_ versus a complete annual mean of the simulated values is presented in Figure 7B. Most regions exhibited a sampling-induced uncertainty (1 SD) within ± 20% of simulated PM_2.5_. Regions of low sampling did not necessarily demonstrate enhanced uncertainty and vice versa. Sampling error of satellite-derived PM_2.5_ is larger in regions influenced by biomass burning, mineral dust, or persistent cloud because of a combination of large seasonal variability and nonrepresentative sampling. We applied the ratio of complete to coincident mean simulated PM_2.5_ to reduce uncertainty from sampling variability.

Validation of this ratio is inhibited by the lack of *in situ* measurements in the regions most significantly affected by intermittent sampling. Statistical comparison over the United States and Canada of noncoincident satellite-derived and *in situ* PM_2.5_ decreases the agreement relative to a coincident comparison (noncoincident: slope = 1.13; *r* = 0.70 vs. coincident: slope = 1.07; *r* = 0.77). This finding supports the need for sampling error correction. Uncertainties derived from both the PM_2.5_ estimate and sampling can vary substantially on the regional scale. Testing the combined uncertainty of ± 25% from both sources reveals that approximately 1 SD of the North American data falls within this overall error envelope. Globally, the population-weighted mean uncertainty in satellite-derived PM_2.5_ is 6.7 μg/m^3^.

### Global ambient PM_2.5_: application to population exposure

**Figure 8:**
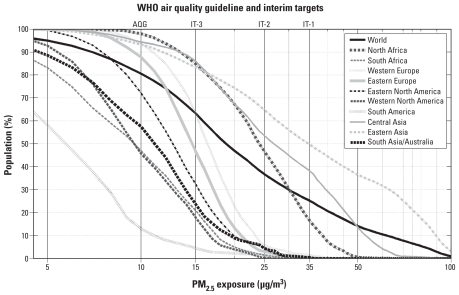
Cumulative distribution of regional, annual mean PM_2.5_ estimated from satellite-derived PM_2.5_ at a resolution of 0.1° × 0.1° for 2001–2006. The top axis identifies WHO AQG and Interim Target (IT) associated with each concentration level. Regions are outlined in Figure 6.

Pope et al. (2009) estimated that a decrease of 10 μg/m^3^ in long-term PM_2.5_ exposure increases life expectancy by 0.61 ± 0.30 years for persons in the United States. We estimated global long-term exposure to ambient PM_2.5_ at a spatial resolution of 0.1° using our satellite-derived values for 2001–2006 and the Gridded Population of the World (GPW; Tobler et al. 1997) data for 2005 from the Socioeconomic Data and Applications Center (GPW version 3; http://sedac.ciesin.columbia.edu/). Figure 8 shows the global and regional distributions of long-term ambient PM_2.5_ exposure; these results are summarized in Table 2. All regions exhibited nonlinear relationships between population and PM_2.5_ concentrations. Eastern and central Asia have the highest levels of PM_2.5_ concentrations, with 38–50% of the regional population exceeding the World Health Organization (WHO) Air Quality Interim Target-1 (WHO 2005) of 35 μg/m^3^. According to the WHO Guidelines, concentrations at this level and higher are associated with an approximately 15% increased risk of mortality, relative to the Air Quality Guideline (AQG) of 10 μg/m^3^. Globally, 80% of the population live in regions that exceed the AQG. These PM_2.5_ estimates should be of considerable value for assessing the chronic health impacts of air pollution, especially in regions with sparse ground-based monitoring.

## Discussion

A major challenge for global epidemiologic studies and assessments of air pollution health impacts is the lack of representative exposure estimates (Cohen et al. 2004). Extensive ground-based monitoring networks exist in some parts of the world, but major portions of the globe are not covered. The situation is especially acute in developing countries with large populations and high pollution levels and where monitoring with traditional ground-based sampling techniques is limited. Although measurements from ground monitors are currently the gold standard for epidemiologic studies, these are not only sparse, but may represent only a small spatial extent in heterogeneous regions (Chen et al. 2006). Satellite observations offer area-integrated values with global coverage, providing valuable additional information for global health studies.

In our study, we produced a satellite-derived climatology of PM_2.5_ concentrations. These estimates should facilitate studies of chronic exposure to particulate matter, similar to those already conducted in Europe and North America (e.g., Beelen et al. 2008; Dockery et al. 1993; Pope et al. 2002, 2009), in regions of the world currently without extensive ground-based monitoring networks. Although a growing number of studies are assessing the impacts of short-term exposure to particulate matter in previously underrepresented regions of the world (e.g., Romieu et al. 2009; Wong et al. 2008), studies of long-term exposure also incorporate impacts related to chronic disease and therefore provide a more comprehensive estimate of overall health effects (Kunzli et al. 2001). Our estimates suggest that a concentration of 20 μg/m^3^ represents the global population-weighted geometric mean of PM_2.5_ and that 80% of the global population resides in locations where ambient concentrations exceed the WHO AQG of 10 μg/m^3^. By applying the satellite-derived PM_2.5_ data set, we also identified global regions and areas of specific concern; half (50%) of the eastern Asian population lives in regions that exceed the WHO Air Quality Interim Target-3 of 35 μg/m^3^ and are therefore at increased risk from air pollution-related health impacts. These results highlight the potential use of satellite aerosol observations to contribute to studies on the chronic effects of air pollution at regional and global scales.

Several notable developments over previous work were included in our estimates. We combined AODs from two satellite instruments (MODIS and MISR) to improve the correlation of AOD versus ground-based PM_2.5_ measurements. Extending the satellite data over 6 years (2001–2006) reduced sampling errors. The unprecedented global spatial resolution of 0.1° × 0.1° retains variation relevant to population distribution. A CTM (GEOS-Chem) was applied to account for aerosol vertical distribution, a key factor affecting the relationship between satellite-retrieved, total column AOD and near-surface PM_2.5_. We found significant spatial agreement between mean coincident satellite-derived and ground-based PM_2.5_ for North America (slope = 1.07; *r* = 0.77; *n* = 1057), as well as evidence of global agreement with noncoincident measurements from published and unpublished data (slope = 0.86; *r* = 0.83; *n* = 244). Notably, this level of agreement with ground-based PM_2.5_ is significantly better than that obtained using a global CTM (GEOS-Chem) without satellite data (Supplemental Material, available online at doi:10.1289/ehp.0901623). Detailed spatial structure in the satellite-derived PM_2.5_ concentrations reflect multiple influences.

We assessed the uncertainty in the satellite-derived product through comparison with independent observations and error propagation. We estimated our coincident satellite-derived PM_2.5_ to be accurate at the 1-SD level to within ± 15% of the satellite-derived value using the relative AOD vertical profile measured by the CALIPSO satellite and the total column AOD from ground-based measurements (AERONET). We found evidence that the effect of nonuniform satellite sampling typically biases annual mean satellite-derived PM_2.5_ by < ± 20% of the satellite-derived value. Larger effects are expected over regions influenced by substantial seasonal variation, by persistent cloud, or for individual, severe pollution events. The overall combined PM_2.5_ uncertainty of ± 25% indicates a mean global, population-weighted uncertainty in PM_2.5_ concentration of 6.7 μg/m^3^.

Additional developments could continue to reduce error in the satellite-derived PM_2.5_ estimates presented here. Increased satellite coverage would reduce sampling concerns and might allow for satellite-derived PM_2.5_ to be applied to studies of temporal or spatiotemporal variation. Further improvements to the AOD retrieval (e.g., Drury et al. 2008) would improve accuracy and reduce sampling bias by reducing data rejection. Simulating the AOD–PM_2.5_ conversion factors at finer spatial resolution would better capture their variability, which is especially important in regions of sharp topographic or emissions gradients. Further development of aerosol speciation capability (e.g., Liu et al. 2007) and satellite-based estimates of additional species, such as NO_2_ (Lamsal et al. 2008), would be valuable to more specifically estimate pollutant concentrations.

## Figures and Tables

**Table 1:** Comparison of coincidently sampled 6-year mean measurements^a^ of daily 24-hr average PM_2.5_ with AOD and satellite-derived PM_2.5_.

Retrievals	Slope^b^	Intercept	*r*	*n*
MODIS AOD	0.020	0.10	0.39	1,218
MISR AOD	0.010	0.11	0.39	353
Average AOD	0.015	0.06	0.44	1,236
Combined AOD	0.017	0.10	0.61	1,057
Satellite-derived PM_2.5_	1.066	−1.75	0.77	1,057

a A minimum of 50 measurements is required for each point.

b Calculated with reduced major-axis linear regression (Hirsh and Gilroy 1984).

**Table 2:** Regional PM_2.5_ statistics, number of observations, and population in excess of WHO AQG and Interim Targets^a^.

Region	Population-weighted statistics [μg/m^3^]				Population- weighted total observations	Population [million people (%)]				
	Mean	SD	GM	GSD		Total	AQG (10 μg/m^3^)	IT-3 (15 μg/m^3^)	IT-2 (25 μg/m^3^)	IT-1 (35 μg/m^3^)
World	27	23	20	2.3	297	6,400	5,100 (81)	4,000 (63)	2,300 (37)	1,600 (25)
Eastern Asia	44	29	34	2.2	270	1,900	1,800 (93)	1,600 (83)	1,200 (65)	940 (50)
Central Asia	31	16	27	1.8	230	1,500	1,400 (94)	1,300 (86)	780 (54)	560 (38)
North Africa	26	10	24	1.5	195	540	530 (98)	450 (85)	250 (47)	8.7 (17)
South Africa	11	6	9	1.9	368	540	240 (47)	110 (21)	7.7 (1.5)	0.1 (0.0)
Eastern Europe	15	5	14	1.4	437	450	400 (88)	210 (47)	14 (3.0)	1.8 (0.4)
South America	7	4	5	2.0	361	400	52 (13)	18 (4.5)	5.3 (1.3)	0.0 (0.0)
Eastern North America	13	5	12	1.5	476	350	250 (72)	110 (32)	17 (4.7)	0.0 (0.0)
South Asia/Australia	12	6	10	1.8	304	310	180 (58)	72 (24)	13 (4.4)	1.3 (0.4)
Western Europe	17	5	16	1.4	311	260	250 (94)	170 (63)	15 (5.9)	3.7 (1.4)
Western North America	11	5	10	1.6	366	120	56 (46)	22 (18)	0.3 (0.2)	0.0 (0.0)

Abbreviations: GM, geometric mean; GSD, geometric standard deviation; IT, interim target.

a Data from WHO (2005). Regions are outlined in Figure 6.
